# Hemorrhagic direct traumatic carotid-cavernous fistula during endoscopic transsphenoidal surgery: intraoperative management and endovascular treatment

**DOI:** 10.3171/2020.4.FocusVid.19918

**Published:** 2020-04-01

**Authors:** Giulia Cossu, Tyler Atkins, Steven D. Hajdu, Francesco Puccinelli, Roy T. Daniel, Mahmoud Messerer

**Affiliations:** Departments of 1 Neurosurgery and; 2 Diagnostic and Interventional Radiology, Lausanne University Hospital, Lausanne, Switzerland

**Keywords:** carotid-cavernous fistula, endoscopy, endovascular treatment, transsphenoidal surgery, video

## Abstract

Carotid-cavernous fistula (CCF) is a rare complication after transsphenoidal surgery with only 10 cases published (Ahuja et al., 1992; Cinar et al., 2013; Cossu et al., 2020; Dolenc et al., 1999; Kalia et al., 2009; Karaman et al., 2009; Kocer et al., 2002; Koitschev et al., 2006; Pigott et al., 1989; Takahashi et al., 1969). Intraoperative findings vary from unrecognized events to life-threatening hemorrhages.

We provide a description of the management of an acute CCF occurring during sphenoidotomy in a patient with pituitary apoplexy. Osteotomy performed in the rostrum resulted in a fracture, which extended toward the intracavernous carotid artery.

Bleeding was managed with mechanical compression. Endovascular treatment allowed closure of the fistula through transarterial coiling and glue. Arterial patency was preserved and the patient had no new neurological deficit.

Drilling should be considered over osteotomy for the anterior sphenoidotomy.

The video can be found here: https://youtu.be/0Me23xIVeNI.

## Transcript

This video is of a surgical complication of a hemorrhagic direct traumatic carotid-cavernous fistula occurring during endoscopic transsphenoidal surgery.

**0:34** Patient is a 40-year-old male with sudden-onset headache and diplopia due to a right sixth nerve palsy, who underwent an MRI, which demonstrated a hemorrhagic suprasellar mass extending into the right cavernous sinus.

With a clear diagnosis of pituitary apoplexy, the patient was offered urgent endoscopic endonasal transsphenoidal resection of the tumor and evacuation of hematoma for decompression of his cavernous sinus.

**0:48** Seen here is our standard positioning of the patient in dorsal decubitus, with the head flexed of 30° and slight turned to the right.

**0:55** The endoscope is introduced here on the left nostril with visualization of the inferior and middle turbinates, followed by lateralization of the middle turbinate and identification of the sphenoid ostia.

**1:03** Exposure of the bone of the sphenoid rostrum upon coagulating and removing the mucosa.

**1:08** The opening of the sphenoid sinus was performed with our standard technique, as we have used in more than 500 cases at our institution. We started with an osteotomy superior to the rostrum and then on each side of the sphenoid ostia.

**1:23** With the onset of brisk bleeding immediately upon removing the osteotome on the right side, the suction was replaced, and visualization is found very difficult.

**1:37** Anesthesia is alerted to the occurrence of the complication as a second suction is introduced to improve visualization.

Suspicion at this time is for either an injury of the internal carotid artery versus a branch of the maxillary artery.

With the next goal to obtain hemostasis, we had several options of hemostatic material possibly including autologous muscle. Interventional radiology was contacted immediately to prepare for emergent angiography once hemostasis was obtained to identify the site of the injury and likely perform definitive treatment.

With better visualization, it was apparent the hemorrhage was coming from the fracture in the bone made by the osteotome, so the primary vessel itself could not be visualized. This eliminated the benefit of the use of autologous muscle.

**2:07** The decision was made to not remove the fragment of sphenoid rostrum in order to instead use cotton balls and the bone to tamponade the bleeding, with complete hemostasis ultimately obtained.

The tails of the cotton balls were secured externally to the patient’s nose, and standard nasal packing was placed.

**2:16** Patient was kept under anesthesia and transported to angiography where this lateral right internal carotid artery injection demonstrates the presence of a direct fistula into the cavernous sinus.

Primary endovascular treatment options for the CC fistula included a transvenous route or a transarterial route with or without carotid sacrifice.

**2:36** Ultimately this fistula was treated with transarterial coiling of the cavernous sinus through the fistula augmented with glue.

**2:56** The nasal packing and cotton balls were removed immediately after the procedure without further bleeding and a last angiographic series confirmed the occlusion of the fistula.

**2:51** Postoperative MR angiography demonstrates continued patency of the right carotid artery.

Final outcome in addition to a patent carotid artery includes no new neurologic deficit. The patient was discharged on postoperative day 10 with a mild improvement in his sixth nerve palsy, with plan for continued surveillance and possible delayed surgery for the adenoma if this remained necessary.

In conclusion, direct carotid-cavernous fistula during transsphenoidal surgery represents a very rare event. Despite the rarity, we have concluded that drilling the sphenoid rostrum is preferable to the use of osteotomes to avoid this serious complication by the mechanism of a posteriorly and superiorly extending fracture as occurred in this case.

**3:24** We examined retrospectively the asymmetric thickness on imaging of the rostrum in our patient seen clearly on this CT reconstructed from the postoperative angiogram. The force required to perform the osteotomy was notably greater on the thicker right side of the sphenoid, thus creating the circumstances for the inadvertent fracture, which is demonstrated here on a second image from the same reconstructed CT, with the arterial contrast extravasating through the bone toward the dense packing material seen against the rostrum. Therefore, we recommend that the thickness of the rostrum be analyzed in all cases with a preoperative CT, especially if an osteotomy is going to be used for the opening.

In the event of an ICA injury, this should be managed initially by mechanical compression at the site of injury along with the use of hemostatic agents. Once the bleeding is controlled, emergency arteriography should be performed to evaluate and definitively treat the injury. Multidisciplinary discussion between the neurosurgeon and endovascular team is necessary to decide the endovascular approach to each unique injury, generally with a goal of preserving ICA patency if possible.
